# Alert system for monitoring changes in patient anatomy during radiation therapy of head and neck cancer

**DOI:** 10.1002/acm2.13342

**Published:** 2021-07-24

**Authors:** Bryan Schaly, Jeff Kempe, Varagur Venkatesan, Sylvia Mitchell, Jeff Chen

**Affiliations:** ^1^ Physics & Engineering Department London Regional Cancer Program London ON Canada; ^2^ Department of Radiation Oncology London Regional Cancer Program London ON Canada; ^3^ Departments of Oncology and Medical Biophysics Western University London ON Canada

**Keywords:** anatomy changes, head and neck cancer, image‐guided radiation therapy

## Abstract

The purpose of this study is to validate a previously developed algorithm for alerting clinicians when to consider re‐CT simulation due to changes in the patient's anatomy during radiation therapy of head and neck cancer. Cone beam computed tomography (CBCT) data were collected prospectively for 77 patients. Each CBCT was mathematically compared to a reference CBCT using the gamma index. We defined the match quality parameter (MQP) as an indicator of CBCT image similarity, where a negative MQP value indicates a poorer CBCT match than the match between the first two CBCT acquired during treatment. If three consecutive MQP values were below a chosen threshold, an “alert” is triggered to indicate action required, for example, possible re‐CT simulation. The timing of image review requests made by the radiation therapists and any re‐CT/re‐plan decisions were documented for each patient's treatment course. The MQP for each patient (including any re‐plans) was calculated in a manner that was blinded from the clinical process. The MQP as a function of fraction number was compared to actual clinical decisions in the treatment progress to evaluate alert system performance. There was a total of 93 plans (including re‐plans) with 34 positives (action required) and 59 negatives (no action required). The sensitivity of the alert system was 0.76 and the false positive rate was 0.37. Only 1 case out of the 34 positive cases would have been missed by both the alert system and our clinical process. Despite the false negatives and false positives, analysis of the timing of alert triggers showed that the alert system could have resulted in seven fewer clinical misses. The alert system has the potential to be a valuable tool to complement human judgment and to provide a quality assurance safeguard to help improve the delivery of radiation treatment of head and neck cancer.

## 1 INTRODUCTION

Radiation therapy of head and neck cancer involves delivering high doses of radiation to a primary tumor or surgical bed along with regional lymph nodes in close proximity to several organs at risk. Daily image‐guided radiation therapy (IGRT) ensures that radiation is delivered to the target volumes with high precision. Many patients tend to experience changes in anatomy during their treatment course which are mainly due to weight loss, tumor response, or setup issues. Re‐planning during the treatment course due to changes in anatomy has the potential to maintain or improve treatment planning goals, that is, target coverage and organ at risk sparing.^1^, ^2^ Deciding whether or not to re‐plan is not a straightforward process because of the amount of staff resources needed. In our previous study,^3^ we developed an anatomy comparison tool using online cone beam computed tomography (CBCT) images acquired during treatment. This was used to develop an algorithm that sends an alert to clinicians to decide whether repeat CT simulation (re‐CT) is necessary. Various strategies to decide when to re‐plan head and neck cancer treatment due to changes in anatomy have been documented. One approach is to order a re‐CT for all patients at some chosen dose level or time point.^4^, ^5^, ^6^ Other methods involve dose calculation using the CBCT images acquired during treatment, where the re‐plan decision can be based on dose to critical organs above some chosen threshold.^6^, ^7^, ^8^, ^9^, ^10^, ^11^ Our method uses CBCT images only, where an alert trigger condition was derived from clinical re‐CT decisions made in our department on previous study cases.^3^ This strategy avoids having to re‐plan all patients since it is not always necessary and most cancer clinics likely do not have the resources to re‐plan every patient. Our method also avoids the need to calculate dose on CBCT images, which have inaccuracies due to CBCT Hounsfield units (HU) as well as limited superior–inferior scanning length.^12^ Our previous study was a proof of concept, where we compared CBCT image pairs for 30 patients retrospectively and it was determined that we could achieve a sensitivity of about 80% with a false positive rate of about 30%. In the current study, we further validate the alert system on a larger patient population and with CBCT data collected in a prospective manner in order to simulate a more realistic clinical setting. We also evaluate the efficiency of the alert system by determining whether the alert system agreed or disagreed with clinician's judgment, which was not performed in the previous study.

## 2 MATERIALS AND METHODS

### 2.1 Patient management

Eighty patients were consented for this ethics approved study, where three patients were excluded due to treatment cancelation for a total of 77 patients. All patients in the study were radical head and neck cancer patients (20 or more fractions), with no selection bias toward prescription dose, staging, diagnosis, site, chemotherapy, post‐surgical, etc. Specific sites are summarized in Table 1. Patients were treated on Varian linear accelerators (TrueBeam or Clinac iX, Varian, Palo Alto, CA, USA) that are equipped with a kilovoltage imaging system mounted 90° to the gantry. This imaging system is capable of acquiring CBCT images as well as planar x‐ray radiographs. Our standard of care is to use daily IGRT, where most patients receive CBCT twice a week and orthogonal x‐ray radiographs on all other days. Daily CBCT is used depending on the case, for example, if the dose to organs at risk such as spinal cord is close to the tolerance dose. In our standard clinical practice, the radiation therapists monitor changes in patient anatomy and send a task to a physicist for image review through Aria (Varian) using their judgment. The physicist reviews the image matches in collaboration with the clinical specialist in radiation therapy (CSRT, Co‐investigator Sylvia Mitchell) and the radiation oncologist if needed. A decision to order re‐CT is made based on the magnitude and duration of the changes. Patients followed our standard clinical practice with no additional imaging, that is, the alert system was not used to make re‐CT decisions and therefore patients did not receive extra imaging dose for study purposes.

**TABLE 1:** Summary of treatment sites

Site	Number
Tongue	19
Tonsil	13
Larynx	13
Oropharynx	9
Salivary gland	8
Oral cavity	6
Hypopharynx	3
Primary unknown	3
Locally advanced skin	2
Floor of mouth	1

### 2.2 CBCT comparison and match quality parameter (MQP)

**FIGURE 1:**
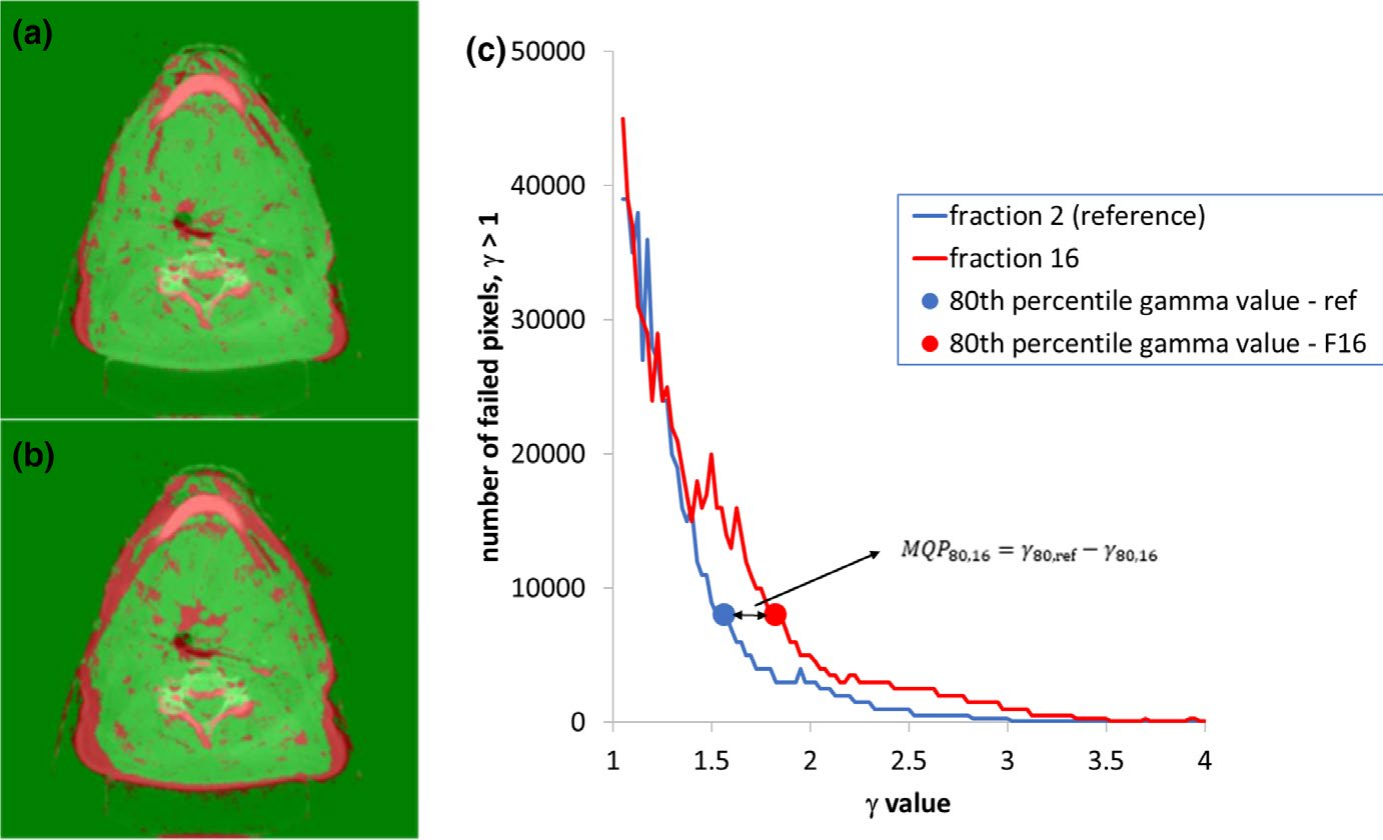
Demonstration of match quality parameter. (a) Gamma map from comparison of cone beam computed tomography (CBCT) on fraction 1 and fraction 2; (b) Gamma map from comparison of CBCT in fraction 1 and fraction 16; and (c) Histograms of γ > 1 from gamma maps in (a) and (b). The match quality parameter value is defined as the difference between 80^th^ percentile values

**FIGURE 2:**
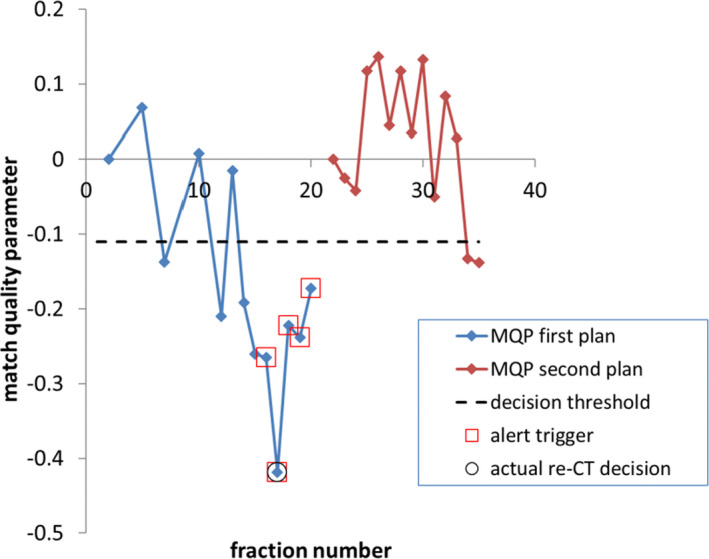
Match quality parameter for a patient that was re‐planned (same patient as in Figure 1)

The method for comparing CBCT image pairs was outlined in our previous work.^3^ The structure set from the plan, the CBCT images, and their corresponding registration files were imported into MIM (MIM Software Inc., Cleveland, OH, USA) which was mainly used for data storage and anonymization. This data were transferred into in‐house developed software for CBCT image comparison. We used the earliest usable CBCT (usually fraction 1) as the reference CBCT. Subsequent usable CBCT images were then compared mathematically to the reference CBCT using the gamma index (global evaluation method)^13^ with criteria of 3 mm distance to agreement (DTA) and 30 HU difference. All gamma maps were generated using the whole image except that gamma values are evaluated from within a mask that is a 1 cm margin around the external contour from the plan, in order to exclude image artifacts that may occur outside of the patient volume. Then from the gamma map, a histogram of the failed pixels (γ > 1) is generated where the gamma value corresponding to the $x^{\text{th}}$ percentile of the histogram is obtained. This is denoted as $\gamma_{x,\text{ref}}$, where “ref” represents the gamma map generated from the first two CBCT image sets acquired during treatment, that is, the reference match. Comparison between the first CBCT and subsequent CBCT then result in gamma maps for fraction i, where the gamma value $\gamma_{x,i}$ is determined. The match quality parameter is defined as

(1) $$MQP_{x,i}=\gamma_{x,\text{ref}}‐\gamma_{x,i}\;,$$

where x is the percentile of the failed pixel histogram obtained from the gamma map generated from the earliest CBCT (${\text{CBCT}}_{\text{ref}}$) and a CBCT acquired at other treatment days, ${\text{CBCT}}_{i}$. The MQP calculation is demonstrated in Figure 1. For this example, the reference match is the CBCT comparison between fraction 1 and 2 (a). The CBCT match between fraction 1 and 16 is shown in Figure 1b, that is, at some later time in the treatment when there was substantial volume change (this patient was re‐planned). The histograms of the pixels that had γ > 1 are shown in Figure 1c, where a poorer match will result in higher gamma values. We determined from our previous work that the optimal percentile was at x=80.^3^ As seen from Figure 1c, there is clear separation between the two histograms at the points corresponding to x=80. Therefore, all MQP calculations in the paper are for x=80 unless otherwise specified. Figure 1 (c) shows that the MQP is a negative value, which means that there are more pixels in the gamma map that fail the criteria compared to the reference match, $\gamma_{80,\text{ref}}$. The MQP was calculated independently after treatment completion for each patient to ensure that the comparison between the alert system and our clinical process was performed in a blinded manner. Figure 2 shows the MQP plotted with fraction number for the same patient shown in Figure 1. The MQP plot shows a downward trend after a few fractions as changes in anatomy get progressively worse. Therefore, the function of the alert system is to use the MQP plot to determine when to recommend image review for possible re‐CT/re‐plan. As described in our previous study, the alert system was trained to trigger an alert within ±3 fractions of the actual re‐CT order date. We defined an alert trigger condition as three consecutive MQP values to be less than a chosen threshold, where −0.11 was obtained from our previous study.^3^ Therefore, all alert triggers for this study were based on this condition in order to validate the alert system in a more realistic clinical setting and using a larger number of patients. Anatomy changes, image review requests by the radiation therapists (RT), and any re‐CT/re‐plan decisions were documented in the patient's treatment record. Then, any alert triggers were compared to what happened clinically (e.g., see Figure 2), so that the sensitivity and timing of the alert system could be evaluated. It should be noted that the MQP by definition is zero for the first match. If there are any re‐plans, then the MQP resets to zero for the new plan(s).

### 2.3 Alert system evaluation

The overall performance of the alert system was evaluated based on sensitivity, false positive rate, and timing when compared to the timing of the RT with respect to flagging the cases to be reviewed. A positive case was defined as the following: (1) patient actually had re‐CT during treatment, (2) cases that should have received consideration for re‐CT based on retrospective review by the project team, and (3) cases where anatomy changes occurred too late for the patient to benefit from a re‐plan. For the positive cases, the re‐CT fraction was defined as the fraction corresponding to the date for which the re‐CT decision was made according to the treatment notes. For positive cases where there was no actual re‐CT, the re‐CT fraction was determined by retrospective review. Negative cases were defined as patients that did not receive re‐CT and were judged as not needing a re‐CT based on retrospective review by the project team (including any re‐plans where re‐CT was not ordered). Therefore, the following definitions were applied:

- True Positive (TP): Alert was triggered within ±3 fractions of the re‐CT fraction.
- True Negative (TN): No alert was triggered for a negative case.
- False Positive (FP): Alert was triggered at any time for a negative case (false alarm).
- False Negative (FN): No alert was triggered within ±3 fractions of the re‐CT fraction (miss).

The TP, TN, FP, and FN were determined with our optimal parameter set, that is, x=80 from Equation (1) and the MQP threshold of −0.11. In order to ensure that the optimal parameter set still holds, receiver operator characteristic (ROC) analysis was performed by first varying the MQP threshold for x=80 and then varying the MQP threshold for different values of x ranging from 50 to 95 in steps of 5. The gamma criteria of 3 mm DTA and 30 HU were not varied since this was investigated previously.^3^ To evaluate the efficiency of the alert system, we compared the timing of the alert system to the timing of the image review requests made by the radiation therapists (RT). For the positive cases, poor timing was indicated if timing difference was outside the ±3 fraction range as defined by TP above. For the negative cases, an alert trigger or image review request made by the RT at any time during treatment indicated poor timing.

## 3 RESULTS

**FIGURE 3:**
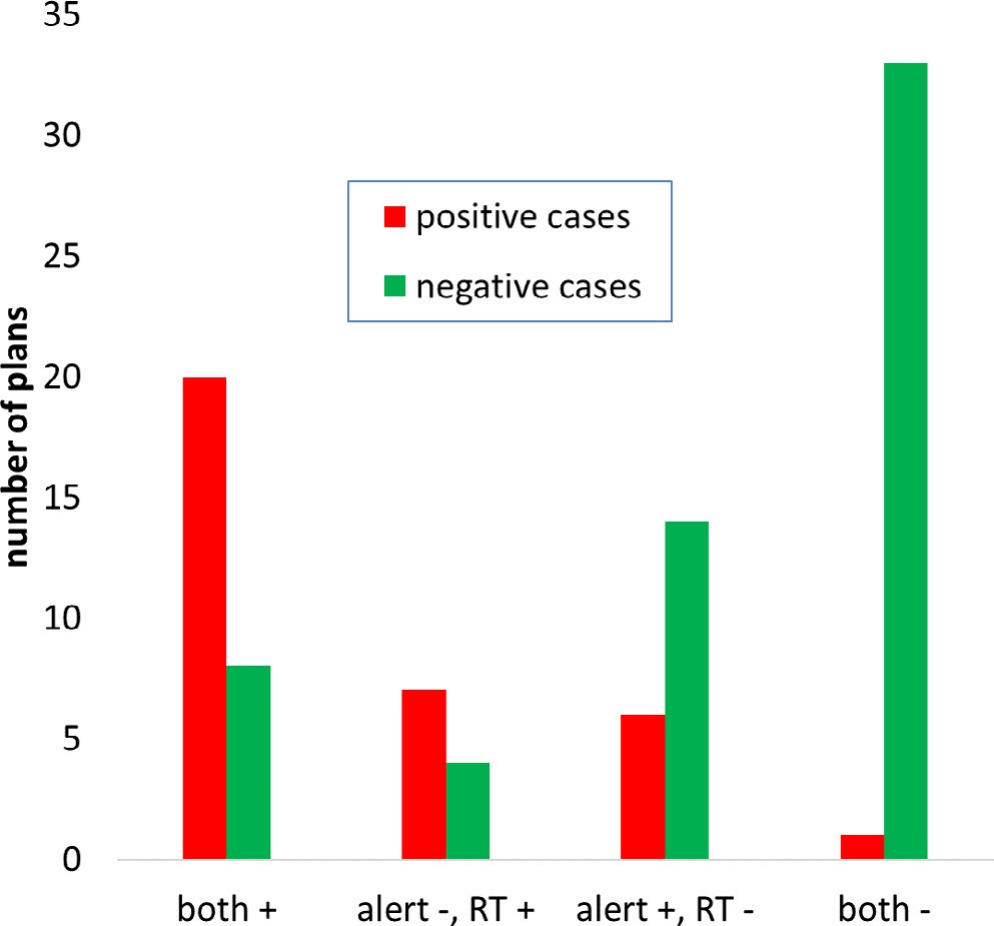
Analysis of alert system timing in comparison with the timing of image review requests made by the radiation therapists

The overall performance of the alert system is summarized in Table 2. Of the 77 patients, there were 93 total plans which includes all the re‐plans (none of the patients had more than one re‐plan). Overall, there were 34 positive cases and 59 negative cases. We achieved a sensitivity of 0.76 and a false positive rate of 0.37 using the alert trigger condition defined earlier. Of the positive cases, 17 patients received re‐CT during treatment, 10 patients were considered potential clinical misses, and 7 patients had anatomical changes that occurred too late for the patient to benefit from a re‐plan. Figure 3 shows the timing of the alert system compared to the timing of the image review tasks assigned by the radiation therapists. The “+” symbol means an alert is triggered or radiation therapist (RT) requests image review within ±3 fractions of the defined re‐CT fraction for positive cases, and at any time for negative cases. The “‐” symbol means the alert trigger or image review request was not within the ±3 fraction range for positive cases or did not occur at all. The radiation therapists (RTs) and the alert system both recommended image review within ±3 fractions of the defined re‐CT fraction in 20 of 34 positive cases. There were eight positive cases where an alert was not triggered. This was mainly due to significant anatomical differences in the reference CBCT match (mainly shoulder position) such that changes in anatomy later in treatment were indistinguishable for the algorithm, that is, the MQP values were greater than the decision threshold. In one case, re‐CT was ordered because of internal tumor changes. This was not detected by the alert system possibly because of the low contrast between gross tumor and surrounding soft tissue in CBCT. However, the alert system had better timing than the RT in six other positive cases. Overall, RT judgment and the alert system missed only one positive case (≈3% of positive cases) when working together.

**TABLE 2:** Summary of patient data and overall alert system performance

Descriptor/metric	Number
Total patients	77
Total plans including re‐plans	93
Total positives	34
Patients received re‐CT	17
Total negatives	59
True positives	26
True negatives	37
False positives	22
False negatives	8

True positive is indicated when an alert is triggered within ±3 fractions of the defined re‐CT fraction in each plan.

**FIGURE 4:**
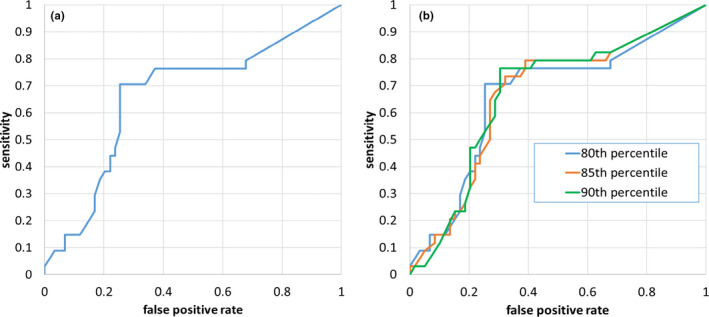
Receiver operator characteristic analysis (ROC). (a) x = 80, where x is defined in Equation (1), (b) ROC comparison between x = 80, 85, and 90

The RT and the alert system both correctly recommended no action in 33 of 59 negative cases. There were 22 false positives made by the alert system which were mainly due to changes in anatomy that were not substantial enough to re‐plan. Overall, the alert system triggered more than the RT for the negative cases. Both the RT and alert system recommended image review for eight negative cases, which would have had the same resource impact on staff. The RT were better at saving resources than the alert system. There were only 4 negative cases where the RT requested image review and the alert system did not, while there were 14 other negative cases where an alert was triggered but the RT did not request image review. Therefore, the alert system with the parameters used is more sensitive than human judgment. One method of decreasing the sensitivity is by lowering the MQP threshold. Figure 4a shows an ROC curve while varying the MQP threshold. For example, lowering the MQP threshold from −0.11 to −0.16 decreases the false positive rate to 0.25 (seven fewer false positives) but with a decrease in sensitivity to 0.71 (two more false negatives). Figure 4b shows a comparison between different percentiles of the failed gamma histogram (the parameter x from Equation (1)). Varying the percentile and MQP threshold did not produce substantially different results. The 90^th^ percentile ROC curve (green) gave a better false positive rate (0.31) with the same sensitivity, while the 85^th^ percentile ROC (orange) curve gave a slightly increased sensitivity (0.79) but with more false positives (0.39).

## 4 DISCUSSION

We have presented a method to determine whether re‐CT is necessary due to anatomy changes during head and neck cancer treatment. We have validated the alert system in a more realistic clinical setting. Therefore, we have confidence that the alert system can be used on patients and further work is needed to make the alert system clinically viable. In the alert system, the patient demographics can be entered by the user manually or taken from the CBCT DICOM data. The structure set from the plan (for the external contour) and the registration file generated from the online match can be imported manually. MQP settings can be chosen by the user according to the desired sensitivity and could be changed for an individual patient, for example, increasing the sensitivity when there is concern about dose to a critical structure. The gamma criteria settings could also be changed though changing the MQP threshold should be sufficient for adjusting the sensitivity and would be easier to implement. In terms of clinical usage, the radiation therapist's role would remain unchanged and their additional workload would be minimal, since the data showed that the alert system and radiation therapists need to be complementary (someone would have to export the CBCT if that was not automated). At our institution, there may be additional workload for the physicists, radiation oncologists, and our CSRT. This is because there is typically more than one alert sent after the initial alert. In the case where a re‐CT is ordered based on the alert, it would make sense to have the alert system turned off until the new plan starts. However, in the case where a re‐plan is not initially needed, getting multiple alerts after the initial alert may become an inconvenience, especially if changes in anatomy are clearly not substantial enough to warrant re‐plan. From the 22 false positives in this data set, 11 cases (50%) would have had an alert trigger sent for every subsequent fraction when a CBCT was acquired after the initial alert (usually twice per week). Six cases would have had more than one additional alert trigger sent but not every fraction, three cases would have had one additional alert sent, and two cases would have had no additional alert triggers. For many of these patients, alerts would have been sent during the last week of treatment which would not take as long to decide whether or not to intervene. To that end, departments can decide to turn off the alerts during the last week of treatment, or for some chosen number of fractions remaining, and rely on the radiation therapists to request plan modifications late in the treatment if needed (e.g., requesting a new immobilization mask). The alert system could also give users the option to decrease the sensitivity during treatment if needed.

The main limitation of the alert system is the false negatives that were caused by significant anatomical differences in the reference match, which was usually the CBCT match between fraction 1 and 4. This resulted in the suppression of the gamma map difference from later CBCT comparisons such that the alert trigger condition could not be met. One way to solve this problem is to use the planning CT as a reference. We chose to use only the CBCT images because fan beam CT and CBCT have different HU values and different scan lengths/number of slices making the gamma comparison more difficult. Mobius CBCT (Varian) has this functionality and uses the electron density instead of HU in the gamma calculation.^14^ Comparison of the alert system performance with Mobius CBCT is a potential area of future work, although much work would be needed to calibrate the pass rates generated by Mobius CBCT to re‐CT decisions made in our department as was performed with the alert system. Another approach by Gros et al^15^ uses the difference between the radial distance in the planning CT and that from the CBCT, which requires preprocessing steps such as defining the external contour in all the CBCT images. The gamma maps and MQP could also be generated from comparisons of a synthetic CT created from CBCT. Work has been performed in this area using machine learning approaches^16^ as well as deformable image registration.^17^ These methods require preprocessing steps to generate the synthetic CT but have the potential for dose calculation. Dose assessment on CBCT was not part of this study but is also a potential area of future work, for example, correlating the MQP values with changes in dose to specific critical organs. Another limitation of the alert system is that there will be always failed pixels no matter how well the online CBCT match is. This “noise” is due to multiple factors including patient rotations that were not accounted for in online image matching, online matching errors, tissue/organ deformation, and imaging artifacts. Investigating the sensitivity of these effects on the gamma maps is also a potential area of future work. Lastly, there is potential to incorporate machine learning methods into this application. Our method is trained to recommend re‐CT based on analyzing trends of a 1‐D pattern of the match quality parameter as derived from gamma comparisons of CBCT images. Perhaps machine learning can be used to discern more patterns in the gamma maps in order to help improve the sensitivity and specificity.

## 5 CONCLUSION

We have developed a cost‐effective alert system to aid in the decision‐making process of when to order re‐CT due to changes in patient anatomy during radiation therapy of head and neck cancer. We have prospectively evaluated the alert system for a larger patient population. Our results have demonstrated that the alert system has the potential to be a valuable complimentary tool to help improve radiation delivery of head and neck cancer.

## CONFLICT OF INTEREST

The authors have no relevant conflict to disclose.

## AUTHOR CONTRIBUTIONS

Bryan Schaly: Principal investigator of the study, performed data collection and analysis, and wrote manuscript. Jeff Kempe: Wrote software for gamma comparisons, performed gamma calculations (blinded from the clinic), and reviewed manuscript. Varagur Venkatesan: Provided clinical insight, helped with data analysis, and reviewed manuscript. Sylvia Mitchell: Recruited and consented patients for the study, provided clinical insight, helped with data analysis, and reviewed manuscript. Jeff Chen: Provided clinical insight, helped with data analysis, and reviewed manuscript.

## References

[1] Castelli J, Simon A, Lafond C, et al. Adaptive radiotherapy for head‐and‐neck cancer. Acta Oncol. 2018;57:1284‐1292. 30289291 10.1080/0284186X.2018.1505053

[2] Bhide SA, Davies M, Burke K, et al. Weekly volume and dosimetric changes during chemoradiotherapy with intensity‐modulated radiation therapy for head and neck cancer: a prospective observational study. Int J Radiat Oncol Biol Phys. 2010;76:1360‐1368. 20338474 10.1016/j.ijrobp.2009.04.005

[3] Schaly B, Kempe J, Venkatesan V, Mitchell S, Battista JJ. Using gamma index to flag changes in anatomy during image‐guided radiation therapy of head‐and‐neck cancer. J Clin App Med Phys. 2017;18:79‐87. 10.1002/acm2.12180 PMC5689936 28901659

[4] Castelli J, Simon A, Louvel G, et al. Impact of head and cancer adaptive radiotherapy to spare the parotid glands and decrease the risk of xerostomia. Radiat Oncol. 2015;10:6. 25573091 10.1186/s13014-014-0318-z PMC4311461

[5] Van Kranen S, Hamming‐Vrieze O, Wolf A, Damen E, van Herk M, Sonke J. Head‐and‐neck margin reduction with adaptive radiation therapy: robustness of treatment plan against anatomy changes. Int J Radiat Oncol Biol Phys. 2016;96:653‐660. 27681762 10.1016/j.ijrobp.2016.07.011

[6] Dewan A, Sharma SK, Dewan AK, et al. Impact of adaptive radiotherapy on locally advanced head‐and‐neck cancer – a dosimetric and volumetric study. Asian Pac J Cancer Prev. 2016;17:985‐992. 27039824 10.7314/apjcp.2016.17.3.985

[7] Belshaw L, Agnew C, Irvine DM, Rooney KP, McGarry CK. Adaptive radiotherapy for head‐and‐neck cancer reduces the requirement for rescans during treatment due to spinal cord dose. Radiat Oncol. 2019;14:189. 31675962 10.1186/s13014-019-1400-3 PMC6825357

[8] Noble DJ, Yeap P, Seah SYK, et al. Anatomical change during radiotherapy of head‐and‐neck cancer and its effect on delivered dose to the spinal cord. Radiother Oncol. 2019;130:32‐38. 30049455 10.1016/j.radonc.2018.07.009 PMC6358720

[9] Vickress JR, Battista J, Barnett R, Yartsev S. Yartsev S Online daily assessment of dose change in head‐and‐neck radiotherapy without dose recalculation. J App Clin. Med Phys. 2018;19:659‐665. 10.1002/acm2.12432 PMC6123138 30084159

[10] Weppler S, Schinkel C, Kirkby C, Smith W. Smith W Lasso logistic regression to derive workflow‐specific algorithm performance requirements as demonstrated for head and neck cancer deformable image registration in adaptive radiation therapy. Phys Med Biol. 2020;65:195013. 32580170 10.1088/1361-6560/ab9fc8

[11] Guidi G, Maffei N, Meduri B, et al. A machine learning tool for re‐planning and adaptive RT: a multicenter cohort investigation. Phys Med. 2016;32:1659‐1666. 27765457 10.1016/j.ejmp.2016.10.005

[12] Yoo S, Yin F. Dosimetric feasibility of cone‐beam CT‐based treatment planning compared to CT‐based treatment planning. Int J Radiat Oncol Phys. 2006;66:1553‐1561. 10.1016/j.ijrobp.2006.08.031 17056197

[13] Low DA, Harms WB, Mutic S, et al. A technique for the quantitative evaluation of dose distributions. Med Phys. 1998;25:656‐661. 9608475 10.1118/1.598248

[14] Lee SS, Min CK, Cho GS, et al. Quantitative evaluation of patient positioning error using CBCT 3D gamma density analysis in radiotherapy Progress. Med Phys. 2017;28:149‐155.

[15] Gros SAA, Xu W, Roeske JC, Choi M, Emami B, Surucu M. A novel surrogate to identify anatomical changes during radiotherapy of head‐and‐neck cancer patients. Med Phys. 2017;44:924‐934. 28019647 10.1002/mp.12067

[16] Borateau A, de Crovoisier R, Largent A, et al. Comparison of CBCT‐based dose calculation methods in head and neck cancer radiotherapy: from Hounsfield unit to density calibration curve to deep learning. Med Phys. 2020;47:4683‐4693. 32654160 10.1002/mp.14387

[17] MacFarlane M, Wong D, Hoover DA, et al. Patient‐specific calibration of cone‐beam computed tomography data sets for radiotherapy dose calculations and treatment plan assessment. J Appl Clin Med Phys. 2018;19:2249‐2257. 10.1002/acm2.12293 PMC5849848 29479821

